# Finite element analysis after rod fracture of the spinal hybrid elastic rod system

**DOI:** 10.1186/s12891-022-05768-x

**Published:** 2022-08-26

**Authors:** Jui-Yang Hsieh, Chen-Sheng Chen, Shao-Ming Chuang, Jyh-Horng Wang, Po-Quang Chen, Yi-You Huang

**Affiliations:** 1 Department of Biomedical Engineering, National Taiwan University, No. 7, Yulu Rd., Wuhu Village, Jinshan Dist., New Taipei City, 20844 Taiwan (R.O.C.); 2 Department of Orthopedic Surgery, National Taiwan University Hospital, Taipei, Taiwan (R.O.C.); 3 Department of Orthopedic Surgery, National Taiwan University Hospital, Jinshan Branch, Taipei, Taiwan (R.O.C.); 4 Department of Physical Therapy and Assistive Technology, National Yang Ming Chiao Tung University, Taipei, Taiwan (R.O.C.)

**Keywords:** Spine hybrid elastic rod, Finite element analysis, Polycarbonate urethane, Nitinol, Dynamic stabilization, Rod fracture

## Abstract

**Background:**

The spinal hybrid elastic (SHE) rod dynamic stabilization system can provide sufficient spine support and less adjacent segment stress. This study aimed to investigate the biomechanical effects after the internal fracture of SHE rods using finite element analysis.

**Methods:**

A three-dimensional nonlinear finite element model was developed. The SHE rod comprises an inner nitinol stick (NS) and an outer polycarbonate urethane (PCU) shell (PS). The fracture was set at the caudal third portion of the NS, where the maximum stress occurred. The resultant intervertebral range of motion (ROM), intervertebral disc stress, facet joint contact force, screw stress, NS stress, and PCU stress were analyzed.

**Results:**

When compared with the intact spine model, the overall trend was that the ROM, intervertebral disc stress, and facet joint force decreased in the implanted level and increased in the adjacent level. When compared with the Ns-I, the trend in the Ns-F decreased and remained nearly half effect. Except for torsion, the PS stress of the Ns-F increased because of the sharing of NS stress after the NS fracture.

**Conclusions:**

The study concluded the biomechanical effects still afford nearly sufficient spine support and gentle adjacent segment stress after rod fracture in a worst-case scenario of the thinnest PS of the SHE rod system.

**Supplementary Information:**

The online version contains supplementary material available at 10.1186/s12891-022-05768-x.

## Background

Supplemental posterior spinal instrumentation is considered the gold standard for the surgical treatment of spinal degenerative diseases, deformities, and fractures [1]. There is still much debate regarding the indications and clinical outcomes of spinal arthrodesis [2]. Common fusion surgery combined decompression osteotomy limits physiological motion and increases the pressure on the adjacent spine. In recent years it has caused a significant inevitable increase in adjacent segment disease, failed back surgery syndrome and mechanical complications [3]. The spinal rod fracture is a serious instrumentation failure and often requires reoperation. It is associated with the older, greater body mass index, larger sagittal rod contour, presence of connectors and crossing thoracolumbar and lumbosacral junctions. Of note, the rod material is another major factor and the rate of rod fracture was significantly higher with cobalt chromium rods than with titanium alloy or stainless-steel rods [4].

Semi-rigid dynamic stabilization systems have been introduced to support the spine and preserve physiological functions [5]. Nevertheless, the existing dynamic systems often cause complications, including bulky implants, complex structures, difficult operation, or new materials without sufficient rigidity [6, 7]. The high rate of early complications and re-revisions made surgeons hesitant to perform non-fusion dynamic stabilizations.

The mechanical structure and material selection should be improved simultaneously for the further development of a perfect dynamic stabilization system [8, 9]. The spinal hybrid elastic (SHE) rod is a semi-rigid pedicle screw-based rod intended for use with universal clinical pedicle screws. This novel construct was created using an inner semi-rigid nitinol stick (NS) and outer flexible polycarbonate urethane (PCU) shell (PS). The SHE rod dynamic stabilization system is used to support the spine and reduce loading across the adjacent segment to limit degeneration while preserving motion.

However, all materials are subjected to fatigue failure due to cyclic loads [10]. To mimic extreme failure conditions after implantation, the rod was set to a rigid fracture. This study aimed to investigate the biomechanical effects on the conditions after the internal fracture of a SHE rod. The hypothesis is that the internal fracture of the SHE rods can still provide nearly sufficient spine support and gentle adjacent segment stress.

## Materials and methods

### Intact lumbar spine model (INT)

This study used a three-dimensional, nonlinear finite element spinal model validated in our previous studies, constructed using ANSYS 14.5 (ANSYS Inc., Canonsburg, PA, USA). A detailed description of the material properties of an INT has been reported [11–13]. The INT includes the vertebrae, intervertebral discs, endplates, posterior bony elements, and all seven ligaments. These intervertebral discs comprised a ground substance, the hyperelastic annulus fibrosus, and the incompressible nucleus pulposus, with 12 double cross-linked fiber layers embedded in the ground substance. The facet joint and the annulus fibrosus were constructed with nonlinear material properties in this model. The facet joint was treated as having a non-contact behavior, and the friction coefficient was set at 0. The initial gap between a pair of facet surfaces was kept 0.5 mm. The parameters of annulus fibrosus were selected as the Young’s modulus of 5.36 MPa and the Poisson’s ratio of 0.45. The nonlinear annulus ground substance was simulated using a hyperelastic Mooney-Rivlin solid model with material constants of C10 = 0.42 and C01 = 0.105. To verify the reliability of the range of motion (ROM) and facet contact force of the INT, they were compared with those of the earlier in vitro tests. It was confirmed that this INT had a stiffness like that of cadaveric lumbar spine studies [14]. The material properties of the spine are listed in Table 1.

**Table 1:** Material properties of the implants used in the finite element model

Dynamic stabilization system		
Titanium alloy pedicle screws	Young’s modulus (MPa)	110,000
	Poisson’s ratio	0.28
PCU shell	Tensile yield	50%
	Young’s modulus (MPa)	68.4
	Poisson’s ratio	0.4
	Yield strength (MPa)	34.2
	Area (mm^2^)	101.13
Nitinol stick	Young’s modulus (MPa)	47,000
	Poisson’s ratio	0.3

*PCU* Polycarbonate urethane

### Implanted model

**Fig. 1:**
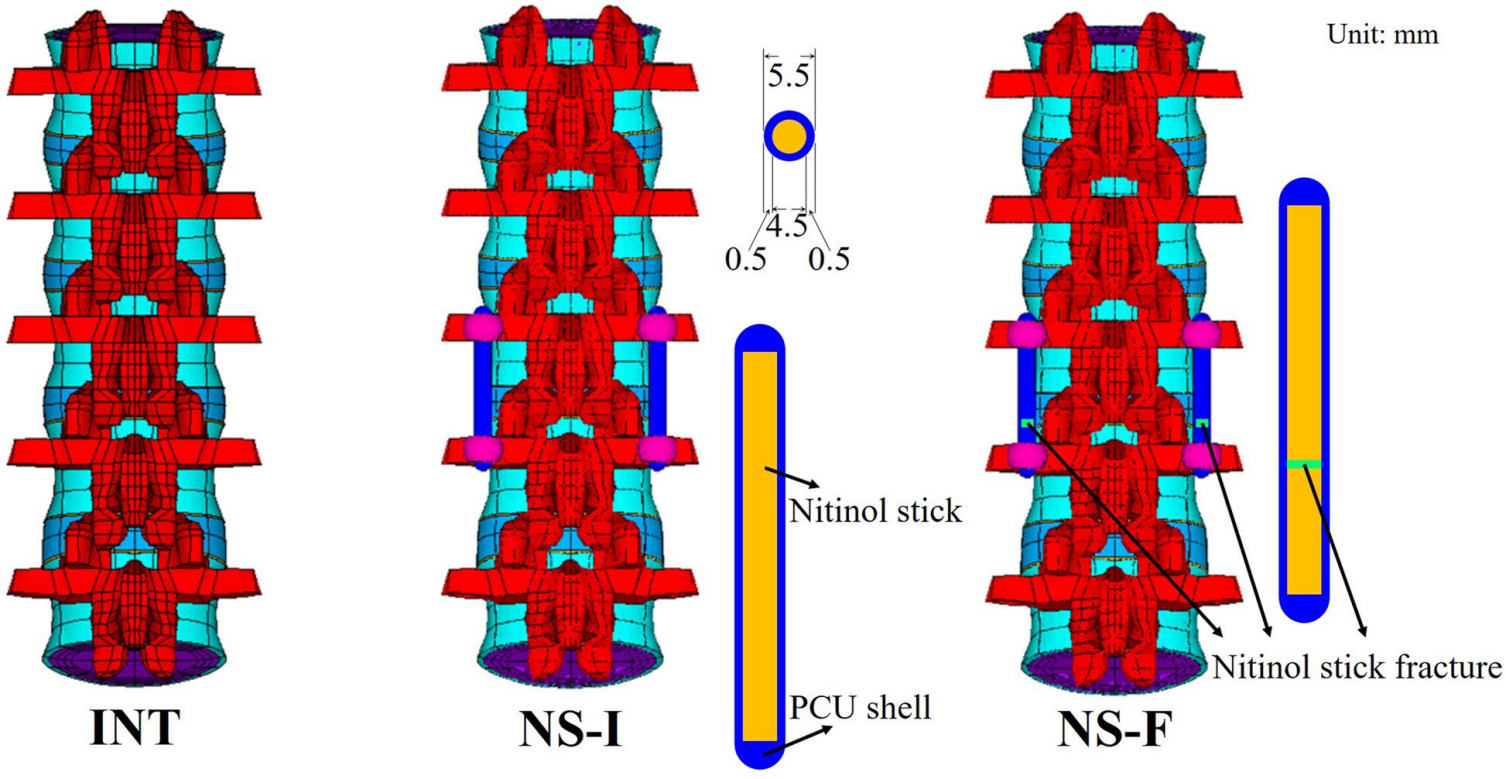
Three finite element spine models were established: intact spine (INT), implanted with a spinal hybrid elastic (SHE) intact rod system (Ns-I), and implanted with a SHE fractured rod system (Ns-F)

The implant system was bilaterally inserted into the L3-L4 level of the INT according to standard surgical procedures. A set of the SHE rod system comprises four conical screws without threads (diameter = 6.4 mm, length = 45 mm) and two SHE rods (diameter = 5.5 mm, length = 30 mm). The SHE rod comprises an inner semi-rigid NS (diameter = 5.5 mm, length = 25 mm) and an outer flexible PS. The material parameters of each element were obtained from previous research [15]. The interface between the pedicle screw and the bone was simulated using total bonded contact elements to provide stable support. No sliding or separation between these edges is allowed under tensile force and compressive force. The interface of the fracture surface of the rod was treated as standard contact to provide semi-rigid fixation for lumbar stability in the finite element model. This type of contact has specified compressive strength but does not resist tensile force. Standard contact elements were also simulated on the interfaces between the pedicle screw/PS and NS/PS. The fracture was set to the distal third portion of the inner NS of the SHE rods where the maximum stress occurred. The SHE rod systems with an intact NS (Ns-I) and fractured NS (Ns-F) were compared with the INT (Fig. 1).

### Boundary and loading conditions

With the model constrained at the bottom of the L5 vertebra, the first step of loading was applying 150 N preload on the superior surface of the L1 vertebra. In the second load step, a higher pure unconstrained moment in 0.36 Nm increments was applied to ensure that the resultant ROM of the L1 to L5 vertebrae would match all four physiological motions. A load was applied with flexion 24°, extension 12.6°, torsion 18.8° and lateral bending 24.8°. The boundary load is the maximum load under convergence. A displacement control method was applied to predict adjacent segment effects after spinal implantation [16]. The cosine function method to determine the ROM used by Hsieh [17] was applied to assess the degree of each motion segment. All values were normalized with respect to intact. The resultant intervertebral ROM and stress of the intervertebral disc and facet joint contact forces were analyzed. Distortion energy theory was applied to the intervertebral discs. The von Mises stress of each model was obtained after applying torque in each direction of the model.

## Results

### Intervertebral ROM

**Fig. 2:**
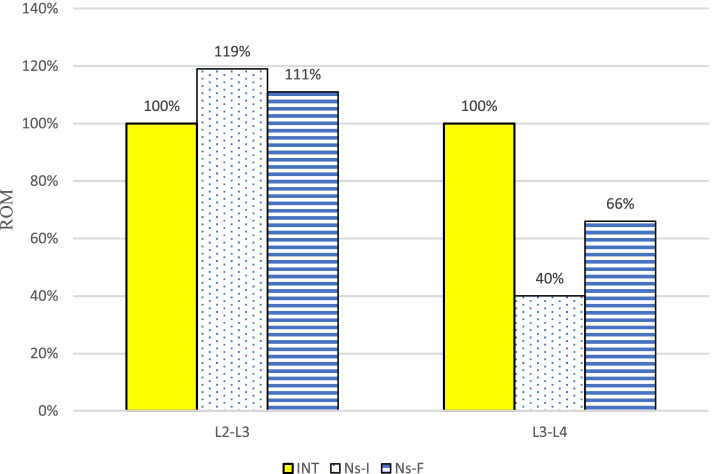
Comparison of the range of motion (ROM) during flexion. The ROM of the fractured nitinol stick (Ns-F) were between those of the intact spine (INT) and intact nitinol stick (Ns-I)

At the implanted L3-L4 level, the ROM in both models decreased compared with the INT. The ROM of the Ns-I and Ns-F decreased to 40 and 66% during flexion, 38 and 64% during extension, 82 and 95% during torsion, and 43 and 76% during lateral bending, respectively. In contrast, the ROM in both models increased compared with the INT at the adjacent L2-L3 level. The ROM of the Ns-I and Ns-F increased to 119 and 111% during flexion, 115 and 109% during extension, 105 and 101% during torsion, and 117 and 108% during lateral bending, respectively. The normalized results are in Fig. 2. After the NS fracture, there was still nearly half the stabilizing effect of the Ns-F, except during torsion, compared with that of the Ns-I. The results with a scale are listed in Table 2.

**Table 2:** Comparison of the intervertebral range of motion (degree) in the finite element models

Model	Level	Flexion	Extension	Torsion	Lateral bending
INT	L2-L3	5.71	3.17	4.08	6.12
	L3-L4	5.71	2.85	4.60	6.17
Ns-I	L2-L3	6.81	3.65	4.28	7.15
	L3-L4	2.28	1.09	3.79	2.63
Ns-F	L2-L3	6.35	3.44	4.10	6.59
	L3-L4	3.79	1.89	4.35	4.67

*INT* Intact model, *Ns-I* Intact nitinol stick, *Ns-F* Fractured nitinol stick

### Intervertebral disc stresses

**Fig. 3:**
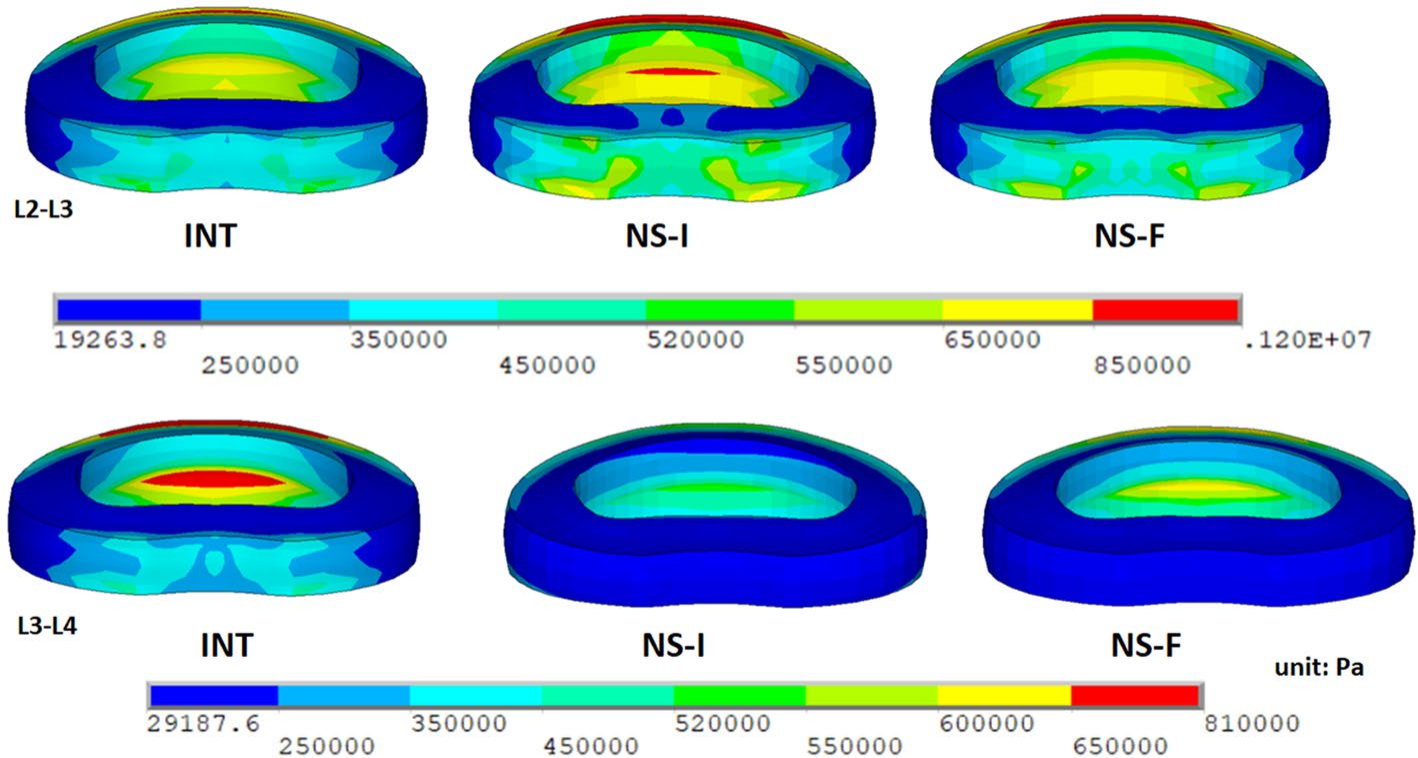
The von Mises stress distribution in the adjacent (L2-L3) and implanted (L3-L4) levels during flexion. The stresses of the fractured nitinol stick (Ns-F) are between those of the intact model (INT) and intact nitinol stick (Ns-I)

**Fig. 4:**
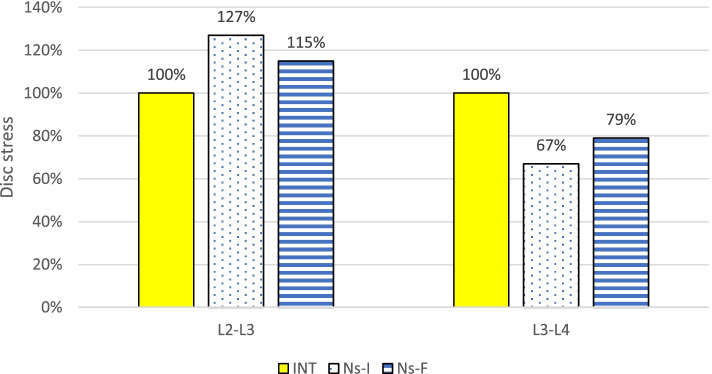
Comparison of the disc stress during flexion. The disc stresses of the fractured nitinol stick (Ns-F) were between those of the intact model (INT) and intact nitinol stick (Ns-I)

At the implanted L3-L4 level, the disc stress in both models decreased compared with the INT. The disc stress of the Ns-I and Ns-F decreased to 67 and 79% during flexion, 57 and 67% during extension, 81 and 96% during torsion, and 45 and 76% during lateral bending, respectively. In contrast, the disc stress in both models increased compared with the INT at the adjacent L2-L3 level. The disc stresses of the Ns-I and Ns-F increased to 127 and 115% during flexion, 114 and 108% during extension, 106 and 100% during torsion, and 124 and 110% during lateral bending, respectively (Fig. 3 and Fig. 4). Overall, the changes in the Ns-F were approximately half of those in the Ns-I (Table 3).

**Table 3:** Comparison of the intervertebral disc stresses (KPa) in the finite element models

Model	Level	Flexion	Extension	Torsion	Lateral bending
INT	L2-L3	893	488	678	1160
	L3-L4	810	414	751	1130
Ns-I	L2-L3	1130	556	722	1440
	L3-L4	543	236	611	512
Ns-F	L2-L3	1030	526	679	1280
	L3-L4	641	277	719	859

*INT* Intact model, *Ns-I* Intact nitinol stick, *Ns-F* Fractured nitinol stick

### Facet joint contact force

There was no facet force during flexion in any of the models. At the implanted L3-L4 level, the facet force in both models decreased compared with the INT. The facet forces of the Ns-I and Ns-F decreased to 67 and 89% during flexion, 57 and 67% during extension, 81 and 96% during torsion, and 45 and 76% during lateral bending, respectively. In contrast, the facet force in both models increased compared with the INT at the adjacent L2-L3 level. The facet forces of the Ns-I and Ns-F increased to 127 and 115% during flexion, 114 and 108% during extension, 106 and 100% during torsion, and 124 and 110% during lateral bending, respectively. Overall, the changes in the Ns-F were approximately half of those in the Ns-I (Table 4).

**Table 4:** Comparison of the facet joint contact forces (N) in the finite element models

Model	Level	Flexion		Extension		Torsion		Lateral bending	
		Left	Right	Left	Right	Left	Right	Left	Right
INT	L2-L3	0	0	94	94	0	336	53	31
	L3-L4	0	0	105	105	0	336	41	9
Ns-I	L2-L3	0	0	117	117	0	374	82	38
	L3-L4	0	0	6	5	0	213	0	0
Ns-F	L2-L3	0	0	106	107	0	344	62	31
	L3-L4	0	0	51	52	0	304	25	0

*INT* Intact model, *Ns-I* Intact nitinol stick, *Ns-F* Fractured nitinol stick

### Screw stresses

**Fig. 5:**
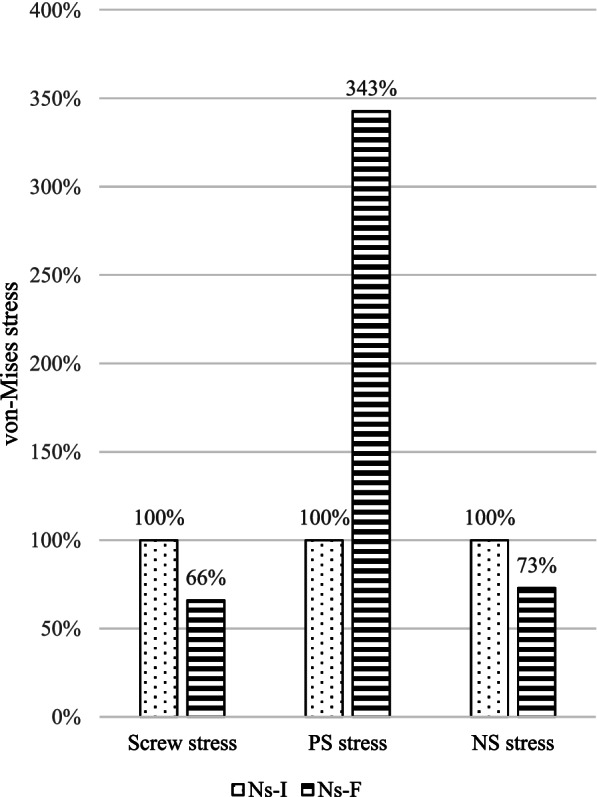
The von Mises stress distribution on the pedicle screw, outer PCU shell (PS) and inner nitinol stick (NS) during flexion. The PS stress of the fractured nitinol stick (Ns-F) was higher than that of intact nitinol stick (Ns-I). In contrast, the screw stress and NS stress were lower in Ns-F group. The outer PS stress was shielded by the inner NS when the NS was intact. Once the inner NS was broken, the screw and NS lost attachment in the Ns-F, and the PS took over the stress. Hence, the NS stress and screw stress decreased, and the PS stress increased

The screw stress of the Ns-F decreased to 66% during flexion, 72% during extension, 41% during torsion, and 60% during lateral bending compared with the Ns-I (Fig. 5). The screw stress of all the models was between 75.6 and 158 MPa, with the maximum stress recorded in the Ns-I during lateral bending (Table 5).

**Table 5:** Comparison of the maximum stress on implants (MPa) in the finite element models

Implant	Motion	Ns-I	Ns-F
Screw	Flexion	139	92.2
	Extension	105	75.6
	Torsion	211	85.5
	Lateral bending	158	94.3
PCU shell	Flexion	4.7	16.1
	Extension	5.1	12.5
	Torsion	17.4	13.3
	Lateral bending	6.8	19.7
Nitinol stick	Flexion	44	32.1
	Extension	39.5	21.5
	Torsion	219	78
	Lateral bending	127	41.2

*Ns-I* Intact nitinol stick, *Ns-F* Fractured nitinol stick, *PCU* Polycarbonate urethane

### PS stress

The PS stress of the Ns-F increased to 343% during flexion, 245% during extension, and 290% during lateral bending compared with the Ns-I (Fig. 5). In contrast, the PS stress decreased by 24% during torsion. The PS stress of all the models was between 4.7 and 19.7 MPa, with the maximum stress recorded in the Ns-F during lateral bending (Table 5).

### NS stress

The NS stress of the Ns-F decreased to 73% during flexion, 54% during extension, 36% during torsion, and 32% during lateral bending compared with the Ns-I (Fig. 5). The NS stress of all the models was between 21.5 and 219 MPa, with the maximum stress recorded in the Ns-I during torsion (Table 5).

## Discussion

This study aimed to investigate the biomechanical effects after the internal fractures of SHE rods. The SHE rod system is a pedicle screw-based construct that incorporates universal rigid fixation technology, combined with the benefits of flexible materials. However, there are intrinsic and extrinsic biomechanical risk factors for any fusion device after fatigue failure [18]. Intrinsic properties include the material type and diameter. Semi-rigid nitinol, the material of the inner SHE rod, is comparable to or superior to titanium rods with regard to biomechanical evidence [19]. Flexible PCU, the material of the outer SHE rod, decreases the stress under the same load by NS stress shielding. Flexion is the most valued physiological movement of the spine owing to its high frequency. The load-controlled findings were the major results, while the other loads were often regarded as minor references. Furthermore, the maximum stress occurred in the caudal third, especially during flexion. Therefore, the fracture was set in the caudal third portion of the NS.

The entire diameter of the SHE rod, including the PS and NS, is the same as that of clinical spinal rods. The universal 5.5-mm diameter can easily promote application and marketing. Increasing the diameter of the NS can increase the rigidity and improve the strength. Increasing the thickness of the PCU can enhance elasticity to provide sufficient support in the case of NS fractures. Currently, the fabrication process hardly makes the PS thinner than 0.5 mm. Our study is the worst-case test scenario for the thinnest PS and thickest NS after rod fracture. The findings confirmed that there was still approximately half of the biomechanical support after the NS fracture. Therefore, when the NS strength is sufficient to cover the physiological conditions, increasing the PS thickness can make the clinical application more reliable.

Other prior studies have proposed the use of Dynesys and PEEK rods to stabilize the lumbar spine dynamically and the protection of adjacent levels by the nonrigid material [5, 6]. The Dynesys consists of a polyethylene terephthalate cord and PCU spacer that are elastic to resist fracture. That is why complications associated with the Dynesys were infection, screw loosening and screw fracture [7]. However, compared to the bulky spacer of Dynesys, the implantation of SHE rods is easier and more intuitive. One concern regarding PEEK rods is their durability and fatigue fracture. The hard surface of PEEK rods is associated with similar clinical risks to metal rods. It has been shown to initiate scratching at the rod-screw interface [20]. That is why PEEK rods need larger diameter and should be locked in specific pedicle screws [6].

**Fig. 6:**
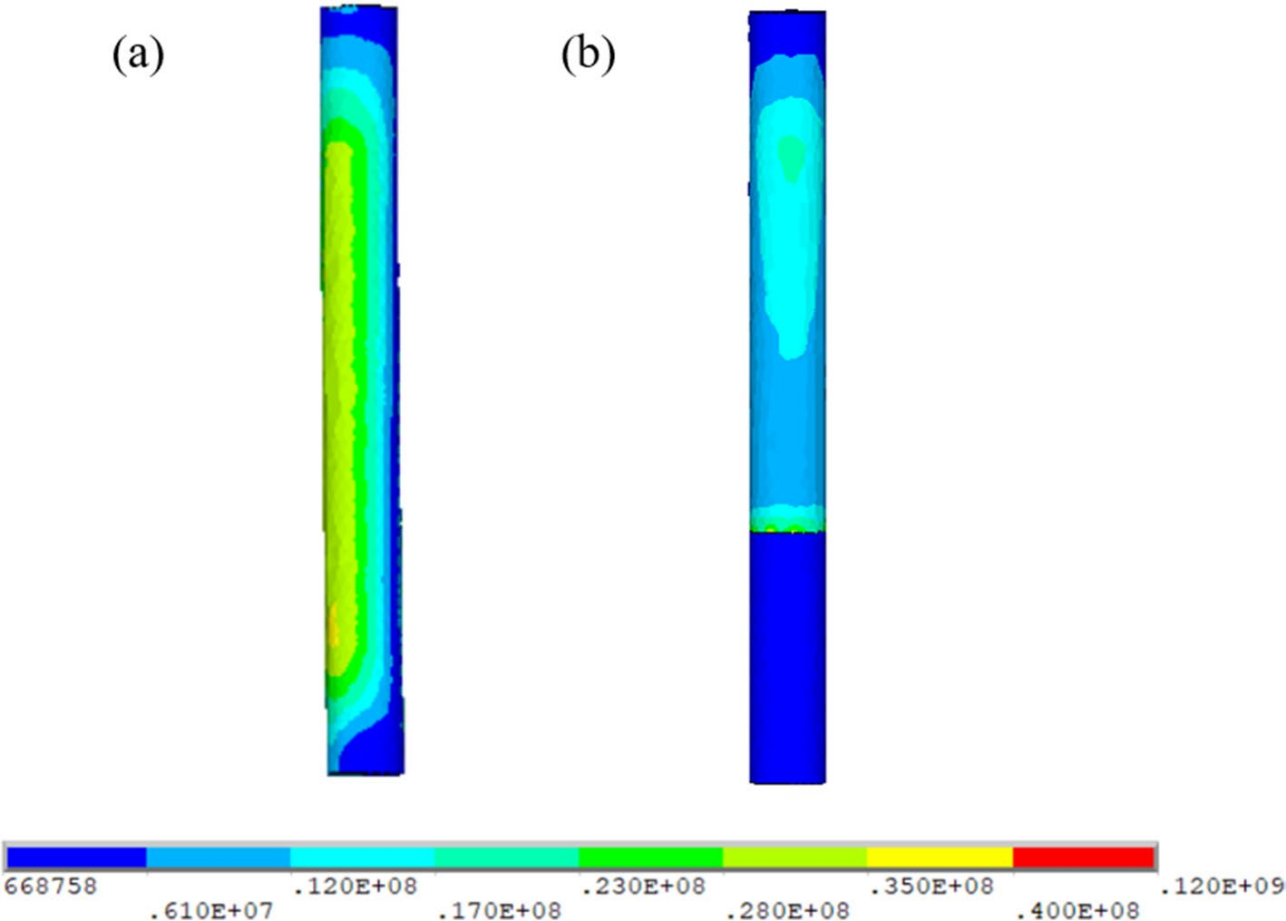
The von Mises stress distribution on the inner intact nitinol stick (a) (Ns-I) and fractured nitinol stick (b) (Ns-F) during flexion. The NS stress markedly increased on the proximal third in Ns-F group. This phenomenon was compatible with the connecting region of the screw and rod

The extrinsic factors can be divided into bending design, notch sensitivity, and cyclic loading. All rods studied in the present model were straight, whereas there may be pre-bent rods in future clinical use. The NS enveloped by the insulated PS was locked by a titanium screw head and nut. This decreases the risk of notch defects and electrochemical corrosion on the surface of the SHE rods and prevents repeated metal strain and fatigue after cyclic loading [21]. Simulated physiological in vivo accelerated 10-year loading had no significant degradative effect on a PCU-nitinol implant [22]. Hence, the hybrid use of PCU-nitinol may lead to the development of durable spinal implants with better clinical compliance (Fig. 6).

The outer PS stress was shielded by the inner NS when the NS was intact. Once the inner NS was broken, the screw and NS lost attachment in the Ns-F, and the PS took over the stress. Hence, the NS stress and screw stress decreased, and the PS stress increased. However, there was an exception while the PS stress decreased during torsion. This was due to the multidirectional torsion shared by the spinal ligaments, intervertebral disc, and facet joint [23]. Theoretically, the stress distributed on the screws and rods returns to the spine after a rod fracture. However, our results showed that screw stress and NS stress decreased but did not disappear. It also indirectly proved that the SHE rods bore partial support from the PS after rod fracture (Fig. 5). Hence, SHE rod fractures differ from common spinal rod fractures.

This study has some limitations. All spinal models were healthy and had no pathological properties, such as degenerative disc diseases, compression fractures, and spondylolisthesis. The INT without decompression was incompatible with the conventional SHE rod implantation surgery. The interface between the pedicle screw and the PS was modeled as always bonded, as were the PS and NS. This study aimed to evaluate the biomechanical effects on the rod, implants contacting the rod, and spinal tissues. Moreover, the interface between the pedicle screw and the bone was assumed to be a union. It was simulated to be bonded contact. Hence, an assessment of the stress distribution of the screw and bone was not reported. Finally, this model demonstrates highly idealized precise implantation and fabrication techniques.

In conclusion, in the worst-case scenario of the thinnest PS, the function remained nearly half after rod fracture of the SHE rod system.

## Supplementary Information

**Additional file 1: Supp.** Table 1**.** The biomechanical analysis of the three finite element models in flexion.

**Additional file 2: Supp.** Table 2The biomechanical analysis of the three finite element models in extension.

**Additional file 3: Supp.** Table 3**.** The biomechanical analysis of the three finite element models in torsion.

**Additional file 4: Supp.** Table 4**.** The biomechanical analysis of the three finite element models in lateral bending.

## Data Availability

The raw data and materials used and analyzed during the current study are provided in a supplementary file. Further detailed datasets are available from the author JYH on reasonable request.

## Abbreviations

SHE: Spinal hybrid elastic
INT: Intact lumbar spine model
NS: Nitinol stick
PCU: Polycarbonate urethane
PS: PCU shell
Ns-I: Intact nitinol stick
Ns-F: Fractured nitinol stick
ROM: Range of motion
