# Supplementary material for: Finite element analysis after rod fracture of the spinal hybrid elastic rod system
Source: BMC Musculoskelet Disord. 2022 Aug 26;23:816. doi: 10.1186/s12891-022-05768-x (PMC9413940; doi:10.1186/s12891-022-05768-x)
Supplement: Supplementary file 1 — Additional file 1: Supp. Table 1. The biomechanical analysis of the three finite element models in flexion. [file 12891_2022_5768_MOESM1_ESM.docx]

**Finite Element Analysis after Rod Fracture of the Spinal Hybrid Elastic Rod System**

**Supp. Table 1.**

The biomechanical analysis of the three finite element models in flexion.

|  | INT | | | Ns-I | | Ns-F | |
| --- | --- | --- | --- | --- | --- | --- | --- |
| Preload (N) | 150 | | 150 | | | 150 | |
| Bending moment (Nm) | 14.4 | | | 18.7 | | 16.9 | |
| Intervertebral range of motion (degree) | | | | | | | |
| L1-L2 | 5.39 | | | 6.43 | | 6.02 | |
| L2-L3 | 5.71 | | | 6.81 | | 6.35 | |
| L3-L4 | 5.71 | | | 2.28 | | 3.79 | |
| L4-L5 | 7.28 | | | 8.60 | | 8.07 | |
| Total | 24.09 | | | 24.12 | | 24.23 | |
| Stress of intervertebral disc (Kpa) | | | | | | | |
| L1-L2 | 1080 | | | 1220 | | 1220 | |
| L2-L3 | 893 | | | 1130 | | 1030 | |
| L3-L4 | 810 | | | 543 | | 641 | |
| L4-L5 | 959 | | | 1200 | | 1100 | |
| Facet contact forces (N) | | | | | | | |
|  | Left | Right | | Left | Right | Left | Right |
| L1-L2 | 0 | 0 | | 0 | 0 | 0 | 0 |
| L2-L3 | 0 | 0 | | 0 | 0 | 0 | 0 |
| L3-L4 | 0 | 0 | | 0 | 0 | 0 | 0 |
| L4-L5 | 0 | 0 | | 0 | 0 | 0 | 0 |
| Stress of screws (MPa) | | | | | | | |
| Maximum | - | | | 139 | | 92.2 | |
| Stress of PCU shell (Mpa) | | | | | | | |
| Maximum | - | | | 4.7 | | 16.1 | |
| Stress of Nitinol stick (Mpa) | | | | | | | |
| Maximum | - | | | 44 | | 32.1 | |
