# Supplementary material for: Finite element analysis after rod fracture of the spinal hybrid elastic rod system
Source: BMC Musculoskelet Disord. 2022 Aug 26;23:816. doi: 10.1186/s12891-022-05768-x (PMC9413940; doi:10.1186/s12891-022-05768-x)
Supplement: Supplementary file 2 — Additional file 2: Supp. Table 2The biomechanical analysis of the three finite element models in extension. [file 12891_2022_5768_MOESM2_ESM.docx]

**Finite Element Analysis after Rod Fracture of the Spinal Hybrid Elastic Rod System**

**Supp. Table 2.**

The biomechanical analysis of the three finite element models in extension.

|  | INT | | | Ns-I | | Ns-F | |
| --- | --- | --- | --- | --- | --- | --- | --- |
| Preload (N) | 150 | | 150 | | | 150 | |
| Bending moment (Nm) | 15.1 | | | 18.4 | | 16.9 | |
| Intervertebral range of motion (degree) | | | | | | | |
| L1-L2 | 3.33 | | | 3.86 | | 3.63 | |
| L2-L3 | 3.17 | | | 3.65 | | 3.44 | |
| L3-L4 | 2.85 | | | 1.09 | | 1.89 | |
| L4-L5 | 3.27 | | | 3.97 | | 3.65 | |
| Total | 12.62 | | | 12.57 | | 12.61 | |
| Stress of intervertebral disc (Kpa) | | | | | | | |
| L1-L2 | 475 | | | 542 | | 507 | |
| L2-L3 | 488 | | | 556 | | 526 | |
| L3-L4 | 414 | | | 236 | | 277 | |
| L4-L5 | 312 | | | 439 | | 407 | |
| Facet contact forces (N) | | | | | | | |
|  | Left | Right | | Left | Right | Left | Right |
| L1-L2 | 76 | 76 | | 95 | 95 | 87 | 87 |
| L2-L3 | 94 | 94 | | 117 | 117 | 106 | 107 |
| L3-L4 | 105 | 105 | | 6 | 5 | 51 | 52 |
| L4-L5 | 102 | 102 | | 129 | 128 | 116 | 116 |
| Stress of screws (MPa) | | | | | | | |
| Maximum | - | | | 105 | | 75.6 | |
| Stress of PCU shell (Mpa) | | | | | | | |
| Maximum | - | | | 5.1 | | 12.5 | |
| Stress of Nitinol stick (Mpa) | | | | | | | |
| Maximum | - | | | 39.5 | | 21.5 | |
