# Supplementary material for: Finite element analysis after rod fracture of the spinal hybrid elastic rod system
Source: BMC Musculoskelet Disord. 2022 Aug 26;23:816. doi: 10.1186/s12891-022-05768-x (PMC9413940; doi:10.1186/s12891-022-05768-x)
Supplement: Supplementary file 3 — Additional file 3: Supp. Table 3. The biomechanical analysis of the three finite element models in torsion. [file 12891_2022_5768_MOESM3_ESM.docx]

**Finite Element Analysis after Rod Fracture of the Spinal Hybrid Elastic Rod System**

**Supp. Table 3.**

The biomechanical analysis of the three finite element models in torsion.

|  | INT | | | Ns-I | | Ns-F | |
| --- | --- | --- | --- | --- | --- | --- | --- |
| Preload (N) | 150 | | 150 | | | 150 | |
| Bending moment (Nm) | 20.9 | | | 31.9 | | 29.6 | |
| Intervertebral range of motion (degree) | | | | | | | |
| L1-L2 | 3.85 | | | 4.13 | | 3.90 | |
| L2-L3 | 4.08 | | | 4.28 | | 4.10 | |
| L3-L4 | 4.60 | | | 3.79 | | 4.35 | |
| L4-L5 | 6.27 | | | 6.62 | | 6.34 | |
| Total | 18.80 | | | 18.82 | | 18.69 | |
| Stress of intervertebral disc (Kpa) | | | | | | | |
| L1-L2 | 683 | | | 740 | | 694 | |
| L2-L3 | 678 | | | 722 | | 679 | |
| L3-L4 | 751 | | | 611 | | 719 | |
| L4-L5 | 927 | | | 1020 | | 949 | |
| Facet contact forces (N) | | | | | | | |
|  | Left | Right | | Left | Right | Left | Right |
| L1-L2 | 0 | 336 | | 0 | 371 | 0 | 342 |
| L2-L3 | 0 | 336 | | 0 | 374 | 0 | 344 |
| L3-L4 | 0 | 336 | | 0 | 213 | 0 | 304 |
| L4-L5 | 0 | 344 | | 0 | 389 | 0 | 354 |
| Stress of screws (MPa) | | | | | | | |
| Maximum | - | | | 211 | | 85.5 | |
| Stress of PCU shell (Mpa) | | | | | | | |
| Maximum | - | | | 17.4 | | 13.3 | |
| Stress of Nitinol stick (Mpa) | | | | | | | |
| Maximum | - | | | 219 | | 78 | |
