# Supplementary material for: Finite element analysis after rod fracture of the spinal hybrid elastic rod system
Source: BMC Musculoskelet Disord. 2022 Aug 26;23:816. doi: 10.1186/s12891-022-05768-x (PMC9413940; doi:10.1186/s12891-022-05768-x)
Supplement: Supplementary file 4 — Additional file 4: Supp. Table 4. The biomechanical analysis of the three finite element models in lateral bending. [file 12891_2022_5768_MOESM4_ESM.docx]

**Finite Element Analysis after Rod Fracture of the Spinal Hybrid Elastic Rod System**

**Supp. Table 4.**

The biomechanical analysis of the three finite element models in lateral bending.

|  | INT | | | Ns-I | | Ns-F | |
| --- | --- | --- | --- | --- | --- | --- | --- |
| Preload (N) | 150 | | 150 | | | 150 | |
| Bending moment (Nm) | 19.1 | | | 23.2 | | 20.9 | |
| Intervertebral range of motion (degree) | | | | | | | |
| L1-L2 | 6.12 | | | 7.25 | | 6.63 | |
| L2-L3 | 6.12 | | | 7.15 | | 6.59 | |
| L3-L4 | 6.17 | | | 2.63 | | 4.67 | |
| L4-L5 | 6.45 | | | 7.79 | | 7.07 | |
| Total | 24.86 | | | 24.82 | | 24.96 | |
| Stress of intervertebral disc (Kpa) | | | | | | | |
| L1-L2 | 1230 | | | 1510 | | 1350 | |
| L2-L3 | 1160 | | | 1440 | | 1280 | |
| L3-L4 | 1130 | | | 512 | | 859 | |
| L4-L5 | 1040 | | | 1320 | | 1160 | |
| Facet contact forces (N) | | | | | | | |
|  | Left | Right | | Left | Right | Left | Right |
| L1-L2 | 21 | 5 | | 30 | 10 | 25 | 7 |
| L2-L3 | 53 | 31 | | 82 | 38 | 62 | 31 |
| L3-L4 | 41 | 9 | | 0 | 0 | 25 | 0 |
| L4-L5 | 55 | 0 | | 61 | 0 | 54 | 0 |
| Stress of screws (MPa) | | | | | | | |
| Maximum | - | | | 158 | | 94.3 | |
| Stress of PCU shell (Mpa) | | | | | | | |
| Maximum | - | | | 6.8 | | 19.7 | |
| Stress of Nitinol stick (Mpa) | | | | | | | |
| Maximum | - | | | 127 | | 41.2 | |
